# Latent profiles of perfectionism and self-compassion: further validation of the tripartite model of perfectionists in South Korea

**DOI:** 10.3389/fpsyg.2025.1570718

**Published:** 2025-05-14

**Authors:** Hyun-joo Park

**Affiliations:** Department of Education, Dongguk University, Seoul, Republic of Korea

**Keywords:** perfectionism, self-compassion, tripartite model, latent profile analysis, South Korea

## Abstract

The tripartite model of perfectionism comprising maladaptive, adaptive, and non-perfectionists, has been consistently supported in the literature. However, the conceptual grounds of the grouping variables which distinguish maladaptive and adaptive perfectionists are relatively weak. Drawing on the robust conceptual intersections of perfectionism and self-compassion and employing Latent Profile Analysis, this cross-sectional study purported to validate the tripartite model of perfectionism with South Korean college students. Data were collected from 375 South Korean college students through an online survey. Participants completed an online survey assessing their levels of perfectionism, self-compassion, depression, psychological distress, self-esteem, and life satisfaction. Latent Profile Analysis revealed three distinct groups of maladaptive perfectionists, adaptive perfectionists, and non-perfectionists, categorized by varying levels of perfectionism and self-compassion. Psychological characteristics of the three groups were illuminated by their mean differences across depression, psychological distress, self-esteem, and life satisfaction. Self-compassion may assist as a key differentiator between adaptive and maladaptive perfectionists, despite their shared perfectionistic tendencies. Implications for the study findings and directions for future research were discussed.

## 1 Introduction

After over 30 years of research, scholars have reached a consensus that perfectionism is a multidimensional personality trait and is characterized by a combination of two superordinate factors, a striving for excessively high standards and a tendency to be overly critical of oneself (Stoeber and Otto, 2006). Stoeber and Otto’s (2006) review was instrumental in conceptualizing perfectionism with these two factors of Perfectionistic Strivings and Perfectionistic Concerns. The Almost Perfect Scale-Revised (APS-R; Slaney et al., 2001) is considered as one of the most widely used measures of perfectionism and comprises three subscales of Standards, Discrepancy, and Order. The Standards subscale represents the Perfectionistic Strivings dimension and is considered the core feature of perfectionism (Slaney et al., 2002). The Discrepancy subscale represents the Perfectionistic Concerns dimension and is defined as the “perception that one consistently fails to meet the high standards one has set for oneself… [that potentially captures] the essential defining negative dimension of the construct” (Slaney et al., 2002, p. 69).

Employing these two higher-order dimensions of Perfectionistic Strivings and Perfectionistic Concerns, a grouping approach has consistently confirmed the existence of perfectionists and non-perfectionists. From a metatheoretical perspective, the grouping approach aligns with the person-centered approach (Spurk et al., 2020), where “the goal of research is to identify distinct groups of individuals with different patterns of variations across a set of variables” (Wong et al., 2012, p. 258). Using cluster analysis and latent profile analysis (LPA), the existence of adaptive and maladaptive perfectionists have been consistently supported in empirical studies (Lee and Park, 2011; Richardson et al., 2014; Suh et al., 2017). Based on the accumulated findings, Stoeber and Otto (2006) proposed the tripartite model of perfectionists of maladaptive perfectionists, adaptive perfectionists, and non-perfectionists. Compared to adaptive perfectionists and nonperfectionists, maladaptive perfectionists displayed significantly higher levels of depression and anxiety (Grzegorek et al., 2004; Lee and Park, 2011) as well as problematic emotion regulation (Richardson et al., 2014). Conversely, adaptive perfectionists exhibited significantly higher levels of self-esteem (Grzegorek et al., 2004), psychological well-being (Park and Jeong, 2015), and happiness and life satisfaction (Suh et al., 2017) than the other two groups.

The tripartite model of perfectionism raises an important question in perfectionism research. Although both maladaptive and adaptive perfectionists maintain high standards for themselves and strive for excellence, the former suffers from harsh self-evaluations and detrimental psychological outcomes, while the latter demonstrates indices of positive psychological functioning. What accounts for this critical difference between these two groups of perfectionists? A key variable to consider is self-compassion, which is defined as “being touched by and open to one’s own suffering, not avoiding or disconnecting from it, generating the desire to alleviate one’s own suffering and to heal oneself with kindness” (Neff, 2003a, p. 87). It also involves experiencing one’s inadequacies and failures with nonjudgmental attitudes and acknowledging that they are part of the common human experience (Neff, 2003b).

The conceptual intersection between perfectionism and self-compassion can be found in the early theoretical articles discussing these two constructs. Hamachek (1978) characterized neurotic (maladaptive) perfectionists as those who are “unable to feel satisfaction because in their own eyes they never seem to do things good enough to warrant that feeling” (p. 27). In contrast, normal (adaptive) perfectionists are “those who derive a very real sense of pleasure from the labors of a painstaking effort and who feel free to be less precise [*perfect*] as the situation permits” (p. 27). Thus, it can be assumed that one of the major differences between maladaptive and adaptive perfectionists lies in how the individual perceives one’s mistakes and failures and whether the individual can accept oneself as an imperfect being. Related to this conceptualization, Neff (2003b) theorized that self-compassion “requires that one does not harshly criticize the self for failing to meet ideal standards” (p. 87). Given that the Perfectionistic Concerns dimension involves excessive self-criticism for failures and the Discrepancy subscale of the APS-R measures “the perception that one consistently fails to meet the high standards one has set for oneself” (Slaney et al., 2002, p. 69), a lack of self-compassion may be a significant contributing factor to the development of maladaptive perfectionism. Furthermore, Neff (2003b) described having self-compassion as “forgiving one’s failings and foibles, respecting oneself as a fully human – and therefore limited and imperfect – being” (p. 87). Therefore, it can be inferred that it is the self-compassion of adaptive perfectionists that contributes to their psychological health and flexibility while striving for high standards and achievement goals.

In this context, Neff (2003a) included perfectionism as a measure of validity in her scale development study of the Self-Compassion Scale (SCS). She hypothesized that self-compassion would be negatively associated with neurotic perfectionism because “those who are more accepting of themselves and their own human fallibility should be less likely to evidence neurotic perfectionism” (p. 228). Conversely, Neff (2003a) speculated that self-compassion might not be significantly associated with adaptive perfectionism because “the compassionate desire for one’s own well-being should mean that one is still motivated to achieve” (p. 228). Consistent with her hypotheses, Neff (2003a) found a significant negative correlation between the SCS score and neurotic perfectionism and a non-significant correlation between the SCS score and adaptive perfectionism.

Empirical research has focused on the role of self-compassion mainly as mediator (Mehr and Adams, 2016; Stoeber et al., 2020) and moderator (Ferrari et al., 2018; Fletcher et al., 2019) between perfectionism and psychological adjustment. However, the literature on perfectionism and self-compassion suggests that self-compassion may have a more intrinsic and critical role in distinguishing between maladaptive and adaptive perfectionists. Researchers have also emphasized the importance of applying clustering methods such as LPA “in a theory-driven way” and ensuring that “the selected variables that form the profiles have a strong conceptual basis” (Spurk et al., 2020). Unfortunately, prior studies on classifying subgroups of perfectionists often lacked a theoretically solid rationale for choosing clustering variables. These studies typically included conscientiousness and neuroticism as clustering variables, simply suggesting the benefits of incorporating higher-order personality variables (Rice et al., 2013; Suh et al., 2017). Given that both theoretical and empirical literature suggest self-compassion be a critical variable in differentiating maladaptive and adaptive perfectionists, investigating how perfectionism and self-compassion form distinct and meaningful latent profiles would significantly strengthen the validity of the tripartite model of perfectionists and fill the gap in the literature by including self-compassion based on robust conceptual grounds.

The unique cultural context of South Korea in understanding the construct of perfectionism has been documented in the literature (Lee and Park, 2011; Rice et al., 2019). The most prominent social phenomenon that influences perfectionism in Korea is the highly competitive educational and school system. It may be worthy understanding a traditional notion in Korea, *hak-bul*, which is defined by Kim (2006) as “the conceptual stratification of society based on an individual’s university degree” (p. 166). It is also a widespread belief in Korea that *hak-bul* plays a critical role in almost every phase of one’s life, including initial job placement and future success (Kim, 2010). Thus, entering a prestigious university is the single most important goal for Korean students and they spend most of their childhood and adolescents setting high standards for oneself and striving to achieve their best academic performance. Unfortunately, Korean students’ pressure to do better does not stop after they enter colleges. The combined effects of global economy downturn and keen competition around decent jobs were likely to make Korean college students feel pressured to be perfectly prepared for the tight job market and to be quite vulnerable for high levels of stress and poor mental health, evidenced by Koreans’ high suicide rates (Jang et al., 2023).

Taken together, the current study purports to validate the tripartite model of perfectionism integrating self-compassion with a sample of South Korean college students using LPA. LPA is “a categorical latent variable modeling approach” (Spurk et al., 2020) that allows researchers to identify latent subgroups of individuals based on a set of variables (Collins and Lanza, 2013). Based on the literature, the author hypothesized that three groups (i.e., maladaptive perfectionists, adaptive perfectionists, and non-perfectionists) would emerge. Two perfectionists groups would be differentiated from non-perfectionists by their levels of Perfectionistic Strivings. Maladaptive perfectionists and adaptive perfectionists would be distinguished by their levels of Perfectionistic Concerns and self-compassion. If a three-class model fits the data, secondly, the researcher hypothesized that: (a) adaptive perfectionists would exhibit the highest levels of psychological well-being (life satisfaction and self-esteem) and the lowest levels of psychological maladjustment (depression and psychological distress) and (b) conversely, maladaptive perfectionists would show the lowest levels of psychological well-being and the highest levels of psychological maladjustment.

## 2 Method

### 2.1 Participants and procedure

A total of 391 undergraduate students at a large university in Seoul, South Korea participated in this study. No concrete guidelines are available to determine a sample size for LPA, but fit indices for mixture models are expected to function appropriately in a sample size of over 300 to 1,000 (Nylund-Gibson and Choi, 2018). After obtaining approval from the Internal Review Board of the institution, participation recruitment messages containing the online survey link were posted on the online class platform. Study participation was entirely voluntary and informed consent was obtained. After excluding 16 cases due to incompleteness and straight-lining, responses from 375 students were used as the final data. Female students consisted of slightly more than half of the sample (*n* = 197, 52.5%). The mean age of the participants was 22.45 years (*SD* = 2.55). More than one-third of the sample were seniors (*n* = 152, 40.5%), followed by juniors (*n* = 113, 30.1%).

### 2.2 Instruments

The Almost Perfect Scale-Revised (APS-R: Slaney et al., 2001) is a widely used measure of trait perfectionism. It comprises 23 items and three subscales of Discrepancy (12 items), Standards (7 items), and Order (4 items). Items are rated on a 7-point Likert-type scale (1 = *strongly disagree* to 7 = *strongly agree*). The APS-R’s psychometric properties have been well documented in numerous studies (e.g., Rice and Ashby, 2007). The Korean version of the APS-R validated by Park (2009) was used. Park (2009) reported adequate levels of internal consistency estimates of the APS-R subscales (αs ranging from 0.76 to 0.88). The coefficient alphas for the Korean APS-R in this study were 0.92 (Discrepancy), 0.83 (Standards), and 0.77 (Order).

The Self-Compassion Scale (SCS) developed by Neff (2003a) is a most commonly used measure of self-compassion, consisting of 26 items across six subscales (Self-Kindness, Common Humanity, Mindfulness, Self-Judgment, Isolation, and Over-Identification). Items are rated on a 5-point Likert-type scale (1 = *almost never* to 5 = *almost always*). Reliability estimates and evidence for validity of the SCS have been well documented (Neff, 2003a). The Korean version of the SCS, translated and validated by Kim et al. (2008), was used. Kim et al. (2008) reported alpha coefficients ranging from 0.74 to 0.81 for the subscales and 0.90 for the total score. In this study, the coefficient alphas for the Korean SCS ranged from 0.77 to 0.85. Based on the recommendations from recent factor analytic studies by Brenner et al. (2017) and Park et al. (2020), self-compassion was operationalized using the sum of the three positive subscales of Self-Kindness, Common Humanity, and Mindfulness.

The Center for Epidemiological Studies-Depression (CES-D: Radloff, 1977) is a 20-item measure assessing depressive symptoms. Respondents rate the frequency of symptoms using a 4-point Likert scale (0 = *not at all* to 3 = *most or all of the time*). The Korean version validated by Chon and Rhee (1992) was used. The Korean CES-D has been found to be a reliable measure with an alpha coefficient of 0.91 (Chon et al., 2001). The alpha coefficient for the Korean CES-D in this study was 0.92.

The Brief Symptom Inventory-18 (BSI-18; Derogatis, 2001) is a shortened version of the 53-item BSI (Derogatis, 1993). It assesses individuals’ psychological distress levels across three symptoms of Somatization, Depression, and Anxiety. Respondents rate the intensity of each symptom on a 5-point Likert scale (0 = *not at all* to 4 = *extremely*). Park et al.’s (2012) validation study of the Korean BSI-18 reported coefficient alphas of 0.73 (Somatization), 0.80 (Depression), and 0.81 (Anxiety). In this study, the alpha coefficients for the Korean BSI-18 were 0.86 (Somatization), 0.88 (Depression), and 0.88 (Anxiety).

The Satisfaction With Life Scale (SWLS; Diener et al., 1985) is a widely used measure of one’s perception of satisfaction with life. Respondents rate items on a 7-point Likert scale (1 = *strongly disagree* to 7 = *strongly agree*). Acknowledged as a robust global measure of life satisfaction, the SWLS exhibits solid psychometric properties (Pavot and Diener, 1993). For the Korean version, Lim et al. (2010) reported satisfactory levels of internal consistency, with alpha coefficients ranging from 0.74 to 0.95. The alpha coefficient for the Korean SWLS in this study was 0.83.

The Rosenberg Self-Esteem Scale (RSE; Rosenberg, 1965) has been widely used as a measure of an individual’s overall self-esteem. Ten items are rated on a 4-point Likert scale (1 = *strongly disagree* to 4 = *strongly agree*). High internal consistency estimates of the RSE have been reported in the U.S. (Goldsmith, 1986). Park and Jeong (2015) reported an alpha coefficient of 0.88 for the Korean RSE. In this study, the coefficient alpha for the Korean RSE was 0.89.

### 2.3 Data analysis

Descriptive statistics and correlation analyses were performed using SPSS 29.0. LPA was conducted with *Mplus* Version 8.2 (Muthén and Muthén, 2017), using a robust maximum likelihood estimator. Full information maximum likelihood was used to generate unbiased parameter estimates. The Auxiliary option (DU3STEP) in *Mplus* was utilized in comparing the means of psychological adjustment variables.

## 3 Results

### 3.1 Descriptive statistics and intercorrelations

Table 1 presents descriptive statistics and intercorrelations of the study variables. Standards subscale of the APS-R was positively correlated with both Discrepancy and Order subscales (*r*s = 0.25, *p* < 0.001). Self-compassion showed a negative association with Discrepancy subscale of the APS-R (*r* = −0.25, *p* < 0.001). Discrepancy subscale was negatively correlated with life satisfaction and self-esteem, and positively associated with depression and psychological distress. On the other hand, self-compassion was positively correlated with life satisfaction and self-esteem, and negatively associated with depression and psychological distress.

**Table 1:** Intercorrelations among the study variables and their means, standard deviations, and possible score ranges.

Variable	1	2	3	4	5	6	7	8	*M*	*SD*	Range
1. Discrepancy	-----								44.23	13.98	12–84
2. High Standards	0.25^***^	-----							34.63	7.16	13–49
3. Order	0.09^***^	0.25^***^	-----						18.01	4.81	4–28
4. Self-Compassion	−0.25^***^	0.08^***^	0.04^**^	-----					38.46	9.30	13–65
5. SWLS	−0.40^***^	0.08^***^	0.08^**^	0.39^***^	-----				21.01	5.70	5–35
6. RSE	−0.62^***^	0.11^***^	0.07^**^	0.45^***^	0.56^***^	-----			29.44	5.65	10–40
7. CES-D	0.55^***^	0.01^***^	−0.09^**^	−0.30^***^	−0.56^***^	−0.69^***^	-----		16.57	10.81	0–60
8. BSI-18	0.49^***^	0.01^***^	−0.08^**^	−0.31^***^	−0.52^***^	−0.66^***^	0.81^***^	-----	14.67	14.33	0–72

Discrepancy, High Standards, and Order are the three subscales of the APS-R; Self-Compassion is the sum of the Self-Kindness, Common Humanity, and Mindfulness subscales of the Self-Compassion Scale; SWLS = Satisfaction With Life Scale; RSE = Rosenberg Self-Esteem; CES-D = Center for Epidemiological Studies - Depression; BSI-18 = Brief Symptom Inventory-18. *N* = 375. ^*^p < 0.05, ^***^p < 0.001.

### 3.2 Latent profile analysis

LPA was conducted on the three subscales of the APS-R (Discrepancy, Standards, and Order) and Self-Compassion. The local independence assumption was modeled by constraining covariances of the residuals for the indicators to zero (Nylund et al., 2007). Each model was tested with 5,000 random starts, and after 100 iterations, 500 optimizations were used in the final stage. Model testing began with a single-class LPA because it was possible that the data contains no distinct underlying profiles. Subsequently, a standard model comparison approach was employed by comparing a *k*-class model against a *k*-1 class model. It was hypothesized that three profiles (maladaptive perfectionists, adaptive perfectionists, and non-perfectionists) would emerge, with up to four profiles possible based on prior research. Means for the indicators were freely estimated, and variances were constrained to be invariant across the profiles.

The fit for the LPA models was evaluated using several indicators. Relatively smaller Bayesian Information Criterion (BIC; Schwarz, 1978) and adjusted Bayesian Information Criterion (aBIC; Sclove, 1987) values suggest better model fit (Nylund et al., 2007). Relatively high entropy values indicate that the identified latent profiles are more discernible. Model comparisons of the *k*-class model with the *k*-1 class model were based on the Lo–Mendell–Rubin (LMR) likelihood ratio test and the Bootstrap Likelihood Ratio Test (BLRT; McLachlan and Peel, 2000). Statistically significant LMR and BLRT values support retaining the *k*-class model as the better fitting model over the *k*-1 class model. Lastly, practical considerations (e.g., the size of the smallest class) and the interpretability of the results were taken into accounts.

The LPA results are presented in Table 2. The BIC values decreased for the one- to three-class models but remained stable for the four-class model, and increased for the five-class model. The entropy values were higher for the four- and five-class models. The LMR and BLRT values for statistical comparisons showed consistency through the three-class model, indicating that the three-class model significantly improved compared to the two-class model. The LMR and BLRT values provided conflicting results for the four- and five-class models. The LMR values were nonsignificant for the four- and five-class models supporting the three-class model, yet the BLRT values were significant for these models. When the LMR and BLRT results conflict, the BIC result and the interpretability of the profiles are generally prioritized (Berlin et al., 2014; Muthén, 2009), suggesting the three-class model was preferrable. In addition, the number of members in the added class for the four- and five-class models were small (*n* = 21 and 20), which is not recommended due to the low power (*n* < 25, Berlin et al., 2014). Furthermore, the four- and five-class models have not been consistently supported in prior research. Considering various statistical fit indices, class sizes, and previous findings and theory, the three-class model was selected as the best model.

**Table 2:** Fit indices for one- to five-class models.

Class	Class	Count	Proportion	BIC	aBIC	Entropy	LMR	*p*	BLRT	*p*
One-class				10605.72	10580.34					
Two-class	1	235	0.63	10591.41	10550.17	0.492	42.51	0.0138	43.95	0.0000
	2	140	0.37							
Three-class	1	132	0.35	10585.91	10528.81	0.547	33.99	0.0255	35.13	0.0000
	2	89	0.24							
	3	154	0.41							
Four-class	1	91	0.24	10585.82	10512.85	0.653	28.76	0.0685	29.73	0.0000
	2	159	0.42							
	3	104	0.28							
	4	21	0.06							
Five-class	1	104	0.28	10596.68	10507.84	0.653	18.17	0.1869	18.78	0.0300
	2	136	0.36							
	3	62	0.17							
	4	53	0.14							
	5	20	0.05							

BIC = Bayesian Information Criteria; aBIC = adjusted Bayesian Information Criteria; LMR = Lo–Mendell–Rubin test; BLRT = Bootstrap Likelihood Ratio test. *N* = 375.

**Figure 1:**
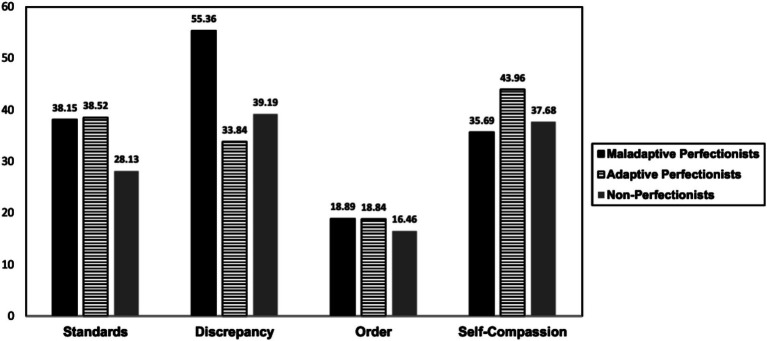
Characteristics of profiles.

For the three-class model, the average class probabilities for the most likely class membership were 0.81, 0.76, and 0.81. The distribution of participants across the three classes were 35.2, 23.7, and 41.1%, respectively. Two classes exhibited significantly higher scores on Standards and comparably higher scores on Order than the third, thus they were classified as perfectionists. Of these two classes of perfectionists, one class scored highest on Discrepancy and lowest on Self-Compassion, while the other displayed the lowest Discrepancy and the highest Self-Compassion scores. The score pattern of the former aligns with maladaptive perfectionists (high Discrepancy and low Self-Compassion) and the latter with adaptive perfectionists (low Discrepancy and high Self-Compassion). The remaining third class was categorized as non-perfectionists, scoring low on both Standards and Order (Figure 1).

To further clarify the characteristics of the three latent classes identified by the LPA, the means of psychological adjustment variables were compared using the Auxiliary option (DU3STEP) in *Mplus*. As presented in Table 3, the overall chi-square tests for mean equality across the four psychological adjustment variables were significant. Compared to maladaptive perfectionists and non-perfectionists, adaptive perfectionists showed the highest levels of life satisfaction and self-esteem and the lowest levels of depression and psychological distress. Maladaptive perfectionists demonstrated the lowest levels of life satisfaction and self-esteem and the highest levels of depression and psychological distress, with non-perfectionists’ scores falling between the other two groups. Wald chi-square tests of mean equality were performed to identify the significant differences among the clusters. The post-hoc chi-square tests were also significant across all three groups on the four psychological adjustment variables (*p*s < 0.017).

**Table 3:** Latent profile means, standard deviations, and Wald chi-square tests of mean equality.

Auxiliary variable	Maladaptive Perfectionists (M)		Adaptive Perfectionists (A)		Non-perfectionists (N)		Global χ^2^	M vs. A	M vs. N	A vs. N
	*M*	*SD*	*M*	*SD*	*M*	*SD*				
SWLS	18.63	0.50	26.42	0.89	20.77	0.55	59.20***	58.84***	7.13***	25.91***
RSE	25.69	0.48	35.52	0.31	29.91	0.59	396.83***	315.04***	25.00***	78.94***
CES-D	23.28	0.98	7.37	0.78	14.29	1.43	186.01***	174.76***	22.37***	19.37***
BSI-18	24.52	1.23	1.56	0.57	7.28	0.93	380.53***	372.12***	155.65***	37.49***

SWLS = Satisfaction With Life Scale; RSE = Rosenberg Self-Esteem; CES-D = Center for Epidemiological Studies - Depression; BSI-18 = Brief Symptom Inventory-18. *N* = 375. ^***^p < 0.017 (Bonferroni correction = 0.05/3; typical *p-*value of 0.05 divided by the number of tests being performed).

## 4 Discussion

Employing the person-centered approach (Spurk et al., 2020), this study identified distinct and meaningful latent groups of maladaptive perfectionists, adaptive perfectionists, and non-perfectionists based on the metrics of perfectionism and self-compassion and further validated the tripartite model of perfectionists with South Korean college students. Consistent with the study hypotheses, the LPA analyses supported three profiles of perfectionism and self-compassion. Although the grouping variables vary, the study results reinforce the robustness of the tripartite model of perfectionists across diverse populations, including South Korea (Lee and Park, 2011; Park and Jeong, 2015), India (Wang et al., 2012), Russia (Wang et al., 2016), and the U.S. (Richardson et al., 2014; Suh et al., 2017).

Maladaptive and adaptive perfectionists shared high levels of Perfectionistic Strivings, yet they were clearly differentiated by their levels of the Perfectionistic Concerns and self-compassion. This finding suggests that self-compassion should be considered as a crucial variable in differentiating maladaptive and adaptive perfectionists. While both groups demonstrated their perfectionistic tendencies through high standards and striving for excellence, adaptive perfectionists’ high levels of self-compassion enable them to treat themselves kindly when faced with mistakes and failures, to embrace their inadequacies and shortcomings, and not to be overly consumed by their experiences. On the other hand, it was the maladaptive perfectionists’ lack of self-compassion that makes them to be harsh and critical toward themselves, hard to forgive their mistakes and failures, and to be overly absorbed in their own experiences. This aligns with Hamachek’s (1978) descriptions of normal and neurotic perfectionists and Neff’s (2003a, 2003b) descriptions of self-compassion in her work. The combination of perfectionism and self-compassion appears to form conceptually meaningful and theoretically coherent groups of perfectionists.

Unhealthy psychological functioning of maladaptive perfectionists with low self-compassion was evidenced by highest levels of depression and psychological distress. This result corroborates previous findings of maladaptive perfectionists’ proneness to psychological distress (Lee and Park, 2011; Rice and Taber, 2019). Conversely, adaptive perfectionists with high self-compassion demonstrated their psychological well-being through elevated levels of life satisfaction and self-esteem. Consistent with the existing research (Park and Jeong, 2015; Suh et al., 2017), these results lend further support to the positive psychological functioning exhibited by adaptive perfectionists. In essence, while setting high standards may not be inherently problematic and can be psychologically healthy, it is the negative dimensions of perfectionism such as Perfectionistic Concerns coupled with a lack of self-compassion that make perfectionists vulnerable to psychological distress.

Self-compassion appears to be a key variable in understanding why some perfectionists are adaptive and others are maladaptive. So far, the role of self-compassion in perfectionism literature has been limited in mediator (Mehr and Adams, 2016; Stoeber et al., 2020) and moderator (Ferrari et al., 2018; Fletcher et al., 2019). Recently, Kawamoto et al. (2023) investigated the mediating role of self-compassion and found that self-compassion explained the differences between maladaptive and adaptive perfectionists across depression, anxiety, and academic distress. Although Kawamoto et al. (2023) primarily focused on the mediating role of self-compassion and used a cut-off score method to create perfectionists groups, their study also underscored the importance of self-compassion in distinguishing between maladaptive and adaptive perfectionists. Continued research efforts are called for to further elucidate how self-compassion operates in perfectionists groups.

Although young generations in South Korea are placed in a culture where they are heavily driven to pursue perfectionism, few studies have been conducted to test if perfectionism scores are comparable between cultures. Rice et al. (2019) found no significant differences on perfectionism between Korean and American college students, but with limitations and cautionary notes. Measurement invariance study results on the self-compassion measure are contradictory in that Taiwanese college students scored the lowest in one study (Neff et al., 2008) whereas Koreans scored the highest in the other (Tóth-Király and Neff, 2021). All in all, more research is needed to understand the cultural implications of perfectionism and self-compassion. Given that the tripartite model of perfectionists incorporating self-compassion was supported in this study, future researchers are encouraged to investigate how adaptive perfectionists in South Korea can manage their perfectionism, thrive in their self-compassion, and maintain healthy functioning in this competitive and self-critical society.

Limitations of the current study should be noted. First, the results were derived from a convenient sample of college students and should be interpreted with limited generalizability. Future studies should consider utilizing more diverse samples of community members and clinical subjects. Second, given the cross-sectional design of this study, researchers are encouraged to replicate the findings using longitudinal designs (e.g., growth mixture modeling) to gain deeper insights into the stability and change within perfectionists groups over time. Third, the study findings were drawn from the participants’ self-report. Future studies are warranted to incorporate diverse measures, such as behavioral and physiological indicators (e.g., stress reactivity, Richardson et al., 2014). Lastly, an ongoing debate exists concerning the structure of self-compassion (one-factor model versus two general factors of self-compassion and self-coldness) (Brenner et al., 2017; Neff et al., 2017). This study adopted two-factor model and operationalized self-compassion as the sum of positive subscales because (a) two-factor model was endorsed as the best-fitting model in South Korea (Park et al., 2020) and (b) self-compassion and self-coldness were found to be differentially related to psychological well-being and distress (Brenner et al., 2018). As more evidence accrue regarding the structure of self-compassion, future researchers could investigate if the study findings are replicated with the different models of self-compassion.

## Data Availability

The raw data supporting the conclusions of this article will be made available by the author, without undue reservation.
